# *O*-Methyl cyclo­laudenol

**DOI:** 10.1107/S1600536809016031

**Published:** 2009-05-07

**Authors:** Nisar Hussain, Habib-ur-Rehman, Masood Parvez

**Affiliations:** a Department of Chemistry, University of Azad Jammu and Kashmir, Muzaffarabad 13100, Pakistan; b Department of Chemistry, The University of Calgary, 2500 University Drive NW, Calgary, Alberta, Canada T2N 1N4

## Abstract

The title compound (systematic name: 3-meth­oxy-24-methyl-9,19-cyclo­lanost-25-ene), C_32_H_54_O, is a triterpenoid which has been isolated from *Skimmia laureola*. The three six-membered rings adopt chair, slightly distorted half-chair and distorted boat conformations, and the five-membered ring adopts an envelope conformation. All the rings are *trans* fused.

## Related literature

For information on *Skimmia laureola*, see: Polunin & Stainton (1984 ▶); Bukingham (1982 ▶); Atta-ur-Rahman *et al.* (2002 ▶). For the structures of closely related compounds, see: Dhaneshwar *et al.* (1986 ▶); Fan *et al.* (2006 ▶). For a description of the Cambridge Structural Database, see: Allen (2002 ▶). For puckering parameters, see: Cremer & Pople (1975 ▶).
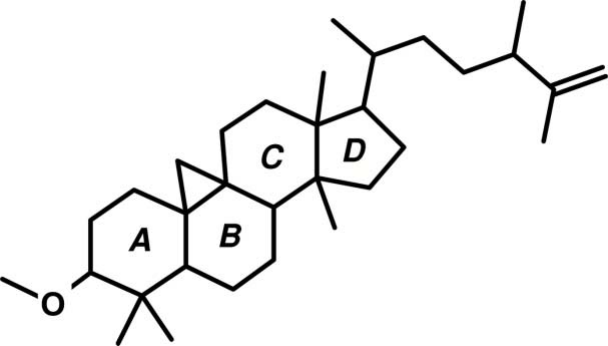


## Experimental

### 

#### Crystal data

C_32_H_54_O
*M* *_r_* = 454.75
Orthorhombic, 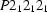

*a* = 6.8812 (2) Å
*b* = 8.5040 (3) Å
*c* = 47.7465 (9) Å
*V* = 2794.02 (14) Å^3^
*Z* = 4
Cu *K*α radiation
μ = 0.46 mm^−1^
*T* = 173 K
0.30 × 0.28 × 0.06 mm

#### Data collection

Bruker APEX2 CCD diffractometer
Absorption correction: multi-scan (*SADABS*; Bruker, 2004 ▶) *T*_min_ = 0.874, *T*_max_ = 0.973
21111 measured reflections
2926 independent reflections
2433 reflections with *I* > 2σ(*I*)
*R*_int_ = 0.099

#### Refinement

*R*[*F* ^2^ > 2σ(*F* ^2^)] = 0.055
*wR*(*F* ^2^) = 0.162
*S* = 1.08
2926 reflections
306 parameters
H-atom parameters constrained
Δρ_max_ = 0.52 e Å^−3^
Δρ_min_ = −0.31 e Å^−3^

### 

Data collection: *APEX2* (Bruker, 2004 ▶); cell refinement: *SAINT* (Bruker, 2004 ▶); data reduction: *SAINT* and *XPREP* (Bruker, 2004 ▶); program(s) used to solve structure: *SHELXS97* (Sheldrick, 2008 ▶); program(s) used to refine structure: *SHELXL97* (Sheldrick, 2008 ▶); molecular graphics: *ORTEP-3 for Windows* (Farrugia, 1997 ▶); software used to prepare material for publication: *SHELXTL* (Sheldrick, 2008 ▶).

## Supplementary Material

Crystal structure: contains datablocks global, I. DOI: 10.1107/S1600536809016031/lh2798sup1.cif

Structure factors: contains datablocks I. DOI: 10.1107/S1600536809016031/lh2798Isup2.hkl

Additional supplementary materials:  crystallographic information; 3D view; checkCIF report

## Figures and Tables

**Fig. 1.**
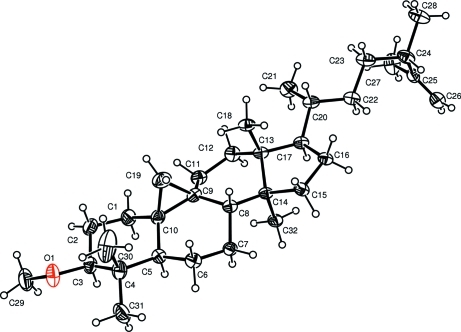
ORTEP-3 (Farrugia, 1997) drawing of (I) with displacement ellipsoids plotted at 30% probability level.

## supplementary crystallographic information

### Comment

*Skimmia laureola* is abundantly found in Northern areas of Pakistan and in Azad Kashmir (Polunin & Stainton, 1984). It finds use in the folk medicine. The strongly aromatic leaves are used in curries or as a flavoring for other foods (Bukingham, 1982). The methanol extract of the plant was subjected to repeated column chromatography to afford a pure triterpene, identified on the basis of spectroscopic studies as *o*-methyl cyclolaudenol, (I) (Atta-ur-Rahman *et al.* 2002). In this paper, we report the crystal structure of (I).

The molecular structure of (I) is presented in Fig. 1. The molecule contains three six-membered rings, A, B and C, a five- membered ring, D and a cyclopropane ring. The ring A adopts a chair conformation. The rings B and C show disotortions due to the *trans*-fused ring D and cyclopropane, exhibiting slightly distorted half-chair and distorted boat conformations, respectively. The puckering parameters (Cremer & Pople, 1975) for the rings A to C are: *Q* = 0.576 (4), 0.503 (4), 0.620 (3) Å, θ = 6.1 (4), 36.7 (5), 71.7 (3)° and φ = 7(4), 90.5 (7), 269.7 (3)°, respectively. Ring D adopts an envelope conformation. All rings are *trans* fused. A search of compounds containing the basic skeleton of (I) in the Cambridge Structural Database (CSD version 5.30; Allen, 2002) yielded only 12 hits, with two compounds closely related to (I), *i.e.*, cimigenol-3-O-β-D-xylopranoside methanol solvate (Fan *et al.*, 2006) and 24-methylene-9,19-cyclolanostan-3β-yl acetate (Dhaneshwar *et al.*, 1986).

### Experimental

The methanol extract of *Skimmia laureola* was subjected to silica-gel column chromatography. The column was eluted with increasing polarities of pet. ether/CHCl_3_. This afforded 4 fractions (PC1—PC4). The fraction PC3 (18 g) obtained by elution with 1 litre pet. ether/CHCl_3_ (7:3) was subjected to column chromatography. The column was successively eluted with 2 litre pet. ether and 3 litre pet. ether/CHCl_3_ (ranging from 9:1 to 7:3) to afford 7 fractions (PC3A—PC3G). The fraction PC3—D (1.4 g) obtained by elution of the column with 500 ml pe t. ether/CHCl_3_ (7:3) was further subjected to column chromatography using 500 ml pe t. ether/CHCl_3_ (7.5:2.5) to afford a pure triterpene, *o*-methyl cyclolaudenol (I) as colourless crystals.

### Refinement

An absolute structure could not be established reliably becuase of insufficient anomalous scattering effects. Therefore, Friedel pairs (2070) were merged. All the H-atoms were visible in the difference Fourier maps, they were included in the refinements at geometrically idealized positions with C—H distances = 0.95 - 1.00 Å, and *U*_iso_ = 1.5 and 1.2 times *U*_eq_ of the methyl and non-methyl C-atoms to which they were bonded. The final difference map was free of chemically significant features.

### Crystal data

**Table:**

C_32_H_54_O	*F*(000) = 1016
*M**_r_* = 454.75	*D*_x_ = 1.081 Mg m^−^^3^
Orthorhombic, *P*2_1_2_1_2_1_	Cu *K*α radiation, λ = 1.54178 Å
Hall symbol: P 2ac 2ab	Cell parameters from 8963 reflections
*a* = 6.8812 (2) Å	θ = 2.8–66.0°
*b* = 8.5040 (3) Å	µ = 0.46 mm^−^^1^
*c* = 47.7465 (9) Å	*T* = 173 K
*V* = 2794.02 (14) Å^3^	Plate, colourless
*Z* = 4	0.30 × 0.28 × 0.06 mm

### Data collection

**Table:**

Bruker APEX2 CCD diffractometer	2926 independent reflections
Radiation source: fine-focus sealed tube	2433 reflections with *I* > 2σ(*I*)
graphite	*R*_int_ = 0.099
φ and ω scans	θ_max_ = 68.0°, θ_min_ = 1.9°
Absorption correction: multi-scan (*SADABS*; Bruker, 2004)	*h* = −7→8
*T*_min_ = 0.874, *T*_max_ = 0.973	*k* = −10→9
21111 measured reflections	*l* = −57→55

### Refinement

**Table:**

Refinement on *F*^2^	Primary atom site location: structure-invariant direct methods
Least-squares matrix: full	Secondary atom site location: difference Fourier map
*R*[*F*^2^ > 2σ(*F*^2^)] = 0.055	Hydrogen site location: inferred from neighbouring sites
*wR*(*F*^2^) = 0.162	H-atom parameters constrained
*S* = 1.08	*w* = 1/[σ^2^(*F*_o_^2^) + (0.091*P*)^2^ + 0.39*P*] where *P* = (*F*_o_^2^ + 2*F*_c_^2^)/3
2926 reflections	(Δ/σ)_max_ = 0.007
306 parameters	Δρ_max_ = 0.52 e Å^−^^3^
0 restraints	Δρ_min_ = −0.31 e Å^−^^3^

### Special details

**Table:**

Geometry. All e.s.d.'s (except the e.s.d. in the dihedral angle between two l.s. planes) are estimated using the full covariance matrix. The cell e.s.d.'s are taken into account individually in the estimation of e.s.d.'s in distances, angles and torsion angles; correlations between e.s.d.'s in cell parameters are only used when they are defined by crystal symmetry. An approximate (isotropic) treatment of cell e.s.d.'s is used for estimating e.s.d.'s involving l.s. planes.
Refinement. Refinement of *F*^2^ against ALL reflections. The weighted *R*-factor *wR* and goodness of fit *S* are based on *F*^2^, conventional *R*-factors *R* are based on *F*, with *F* set to zero for negative *F*^2^. The threshold expression of *F*^2^ > σ(*F*^2^) is used only for calculating *R*-factors(gt) *etc*. and is not relevant to the choice of reflections for refinement. *R*-factors based on *F*^2^ are statistically about twice as large as those based on *F*, and *R*- factors based on ALL data will be even larger.

### Fractional atomic coordinates and isotropic or equivalent isotropic displacement parameters (Å^2^)

**Table:**

	*x*	*y*	*z*	*U*_iso_*/*U*_eq_	
O1	0.1899 (5)	0.0137 (4)	0.74062 (6)	0.0764 (9)	
C1	0.3668 (6)	0.3579 (5)	0.69846 (8)	0.0572 (10)	
H1A	0.4315	0.4572	0.7040	0.069*	
H1B	0.2418	0.3845	0.6893	0.069*	
C2	0.3299 (6)	0.2576 (5)	0.72419 (7)	0.0568 (10)	
H2A	0.4552	0.2323	0.7333	0.068*	
H2B	0.2495	0.3172	0.7377	0.068*	
C3	0.2278 (5)	0.1084 (5)	0.71638 (7)	0.0492 (9)	
H3	0.1000	0.1371	0.7078	0.059*	
C4	0.3393 (6)	0.0070 (5)	0.69525 (8)	0.0551 (9)	
C5	0.3954 (4)	0.1124 (4)	0.67005 (7)	0.0378 (7)	
H5	0.2685	0.1453	0.6617	0.045*	
C6	0.5016 (5)	0.0263 (4)	0.64634 (7)	0.0464 (8)	
H6A	0.6274	−0.0143	0.6533	0.056*	
H6B	0.4226	−0.0644	0.6400	0.056*	
C7	0.5368 (5)	0.1368 (4)	0.62185 (7)	0.0413 (7)	
H7A	0.4116	0.1830	0.6158	0.050*	
H7B	0.5910	0.0766	0.6059	0.050*	
C8	0.6778 (4)	0.2694 (4)	0.62975 (6)	0.0358 (7)	
H8	0.8029	0.2161	0.6346	0.043*	
C9	0.6145 (4)	0.3558 (4)	0.65634 (7)	0.0386 (7)	
C10	0.4960 (5)	0.2668 (4)	0.67803 (7)	0.0440 (8)	
C11	0.5894 (5)	0.5305 (4)	0.65498 (8)	0.0495 (9)	
H11A	0.6105	0.5725	0.6741	0.059*	
H11B	0.4521	0.5517	0.6501	0.059*	
C12	0.7169 (5)	0.6262 (4)	0.63488 (8)	0.0477 (8)	
H12A	0.6320	0.6915	0.6228	0.057*	
H12B	0.8000	0.6981	0.6460	0.057*	
C13	0.8457 (4)	0.5251 (3)	0.61633 (7)	0.0356 (7)	
C14	0.7264 (4)	0.3812 (4)	0.60557 (6)	0.0340 (7)	
C15	0.8613 (5)	0.3116 (4)	0.58314 (7)	0.0423 (8)	
H15A	0.7849	0.2573	0.5685	0.051*	
H15B	0.9528	0.2353	0.5916	0.051*	
C16	0.9722 (4)	0.4519 (3)	0.57066 (7)	0.0385 (7)	
H16A	0.9337	0.4684	0.5509	0.046*	
H16B	1.1140	0.4324	0.5713	0.046*	
C17	0.9196 (4)	0.5982 (4)	0.58850 (7)	0.0376 (7)	
H17	0.8054	0.6495	0.5793	0.045*	
C18	1.0218 (4)	0.4705 (4)	0.63395 (7)	0.0453 (8)	
H18A	1.1016	0.5618	0.6389	0.068*	
H18B	1.0999	0.3962	0.6230	0.068*	
H18C	0.9756	0.4190	0.6511	0.068*	
C19	0.7061 (6)	0.2835 (7)	0.68323 (9)	0.0694 (12)	
H19A	0.7885	0.1889	0.6810	0.083*	
H19B	0.7477	0.3565	0.6982	0.083*	
C20	1.0867 (5)	0.7216 (4)	0.58896 (7)	0.0425 (8)	
H20	1.2018	0.6733	0.5984	0.051*	
C21	1.0257 (6)	0.8684 (4)	0.60564 (9)	0.0600 (10)	
H21A	0.9018	0.9081	0.5984	0.090*	
H21B	1.1256	0.9499	0.6037	0.090*	
H21C	1.0110	0.8408	0.6255	0.090*	
C22	1.1457 (5)	0.7682 (4)	0.55941 (8)	0.0480 (8)	
H22A	1.1622	0.6712	0.5482	0.058*	
H22B	1.0381	0.8290	0.5509	0.058*	
C23	1.3292 (5)	0.8639 (5)	0.55714 (9)	0.0565 (10)	
H23A	1.4376	0.8030	0.5654	0.068*	
H23B	1.3136	0.9609	0.5684	0.068*	
C24	1.3838 (5)	0.9098 (4)	0.52733 (9)	0.0519 (9)	
H24	1.3963	0.8103	0.5163	0.062*	
C25	1.2306 (5)	1.0072 (4)	0.51349 (7)	0.0461 (8)	
C26	1.1536 (7)	0.9661 (6)	0.48956 (9)	0.0729 (12)	
H26A	1.0581	1.0309	0.4810	0.087*	
H26B	1.1933	0.8713	0.4807	0.087*	
C27	1.1717 (8)	1.1550 (5)	0.52792 (10)	0.0734 (13)	
H27A	1.2838	1.2256	0.5293	0.110*	
H27B	1.1243	1.1302	0.5468	0.110*	
H27C	1.0681	1.2065	0.5172	0.110*	
C28	1.5849 (6)	0.9932 (6)	0.52652 (11)	0.0741 (13)	
H28A	1.6225	1.0121	0.5070	0.111*	
H28B	1.6824	0.9262	0.5356	0.111*	
H28C	1.5765	1.0937	0.5365	0.111*	
C29	0.0543 (8)	0.0814 (7)	0.75927 (9)	0.0874 (16)	
H29A	0.1197	0.1600	0.7710	0.131*	
H29B	−0.0011	−0.0008	0.7712	0.131*	
H29C	−0.0499	0.1320	0.7486	0.131*	
C30	0.5252 (9)	−0.0656 (7)	0.70874 (11)	0.108 (2)	
H30A	0.6043	0.0183	0.7170	0.162*	
H30B	0.6008	−0.1204	0.6943	0.162*	
H30C	0.4874	−0.1403	0.7234	0.162*	
C31	0.2096 (9)	−0.1271 (6)	0.68548 (10)	0.0895 (17)	
H31A	0.1750	−0.1933	0.7015	0.134*	
H31B	0.2790	−0.1904	0.6716	0.134*	
H31C	0.0911	−0.0839	0.6771	0.134*	
C32	0.5379 (4)	0.4347 (4)	0.59071 (7)	0.0451 (8)	
H32A	0.4765	0.3441	0.5816	0.068*	
H32B	0.5695	0.5143	0.5766	0.068*	
H32C	0.4484	0.4796	0.6045	0.068*	

### Atomic displacement parameters (Å^2^)

**Table:**

	*U*^11^	*U*^22^	*U*^33^	*U*^12^	*U*^13^	*U*^23^
O1	0.104 (2)	0.079 (2)	0.0468 (15)	0.008 (2)	0.0154 (15)	0.0182 (15)
C1	0.069 (2)	0.053 (2)	0.050 (2)	−0.005 (2)	0.0142 (18)	−0.0158 (17)
C2	0.054 (2)	0.076 (3)	0.0411 (19)	0.004 (2)	−0.0014 (15)	−0.0095 (19)
C3	0.0523 (19)	0.058 (2)	0.0376 (17)	0.0048 (18)	−0.0023 (14)	0.0070 (16)
C4	0.071 (2)	0.049 (2)	0.045 (2)	0.0100 (19)	0.0023 (16)	0.0086 (17)
C5	0.0321 (14)	0.0380 (17)	0.0434 (17)	0.0078 (13)	−0.0011 (12)	0.0029 (14)
C6	0.0471 (18)	0.0357 (17)	0.056 (2)	0.0041 (15)	0.0049 (15)	−0.0080 (15)
C7	0.0404 (16)	0.0389 (17)	0.0446 (18)	−0.0031 (14)	0.0065 (13)	−0.0127 (14)
C8	0.0280 (14)	0.0333 (15)	0.0460 (17)	0.0068 (12)	−0.0022 (12)	−0.0049 (13)
C9	0.0362 (15)	0.0389 (17)	0.0407 (17)	0.0089 (13)	−0.0057 (12)	−0.0058 (14)
C10	0.0446 (17)	0.0473 (19)	0.0400 (17)	0.0010 (15)	0.0020 (13)	−0.0075 (15)
C11	0.0406 (17)	0.052 (2)	0.056 (2)	0.0047 (16)	0.0022 (14)	−0.0175 (17)
C12	0.0490 (18)	0.0367 (17)	0.057 (2)	0.0100 (15)	0.0037 (15)	−0.0090 (15)
C13	0.0289 (14)	0.0287 (15)	0.0492 (18)	0.0049 (12)	−0.0049 (11)	−0.0052 (13)
C14	0.0241 (13)	0.0333 (16)	0.0445 (16)	0.0039 (12)	−0.0024 (11)	−0.0073 (13)
C15	0.0362 (16)	0.0361 (17)	0.055 (2)	−0.0020 (13)	0.0096 (13)	−0.0099 (15)
C16	0.0323 (14)	0.0326 (16)	0.0506 (18)	−0.0003 (13)	0.0013 (12)	−0.0031 (13)
C17	0.0299 (14)	0.0327 (16)	0.0501 (18)	0.0061 (12)	−0.0066 (12)	−0.0015 (14)
C18	0.0351 (16)	0.0427 (17)	0.058 (2)	−0.0027 (14)	−0.0141 (14)	0.0021 (15)
C19	0.045 (2)	0.094 (3)	0.070 (3)	0.001 (2)	−0.0110 (18)	−0.013 (2)
C20	0.0397 (16)	0.0289 (16)	0.059 (2)	0.0010 (13)	−0.0102 (14)	0.0001 (15)
C21	0.075 (2)	0.0304 (17)	0.075 (3)	−0.0032 (18)	0.001 (2)	−0.0058 (17)
C22	0.0391 (17)	0.0407 (18)	0.064 (2)	−0.0028 (14)	−0.0044 (15)	0.0014 (16)
C23	0.0426 (18)	0.047 (2)	0.080 (3)	−0.0077 (17)	−0.0171 (17)	0.0151 (19)
C24	0.0399 (17)	0.0351 (17)	0.081 (3)	−0.0040 (15)	0.0086 (16)	−0.0033 (18)
C25	0.0441 (17)	0.047 (2)	0.0472 (19)	−0.0039 (16)	0.0125 (14)	−0.0014 (16)
C26	0.069 (3)	0.088 (3)	0.061 (3)	−0.021 (3)	0.006 (2)	0.005 (2)
C27	0.092 (3)	0.052 (2)	0.076 (3)	0.026 (2)	0.019 (2)	0.006 (2)
C28	0.046 (2)	0.062 (3)	0.114 (4)	−0.016 (2)	0.005 (2)	0.007 (3)
C29	0.102 (4)	0.106 (4)	0.055 (3)	−0.007 (3)	0.027 (2)	0.011 (3)
C30	0.151 (5)	0.108 (4)	0.066 (3)	0.082 (4)	0.001 (3)	0.027 (3)
C31	0.131 (4)	0.065 (3)	0.073 (3)	−0.041 (3)	0.041 (3)	−0.007 (2)
C32	0.0295 (14)	0.057 (2)	0.0486 (18)	−0.0005 (14)	−0.0070 (13)	0.0032 (16)

### Geometric parameters (Å, °)

**Table:**

O1—C29	1.413 (6)	C16—H16B	0.9900
O1—C3	1.434 (4)	C17—C20	1.557 (4)
C1—C2	1.517 (5)	C17—H17	1.0000
C1—C10	1.531 (5)	C18—H18A	0.9800
C1—H1A	0.9900	C18—H18B	0.9800
C1—H1B	0.9900	C18—H18C	0.9800
C2—C3	1.498 (6)	C19—H19A	0.9900
C2—H2A	0.9900	C19—H19B	0.9900
C2—H2B	0.9900	C20—C22	1.521 (5)
C3—C4	1.534 (5)	C20—C21	1.539 (5)
C3—H3	1.0000	C20—H20	1.0000
C4—C31	1.521 (6)	C21—H21A	0.9800
C4—C5	1.550 (5)	C21—H21B	0.9800
C4—C30	1.560 (6)	C21—H21C	0.9800
C5—C10	1.532 (5)	C22—C23	1.506 (5)
C5—C6	1.534 (4)	C22—H22A	0.9900
C5—H5	1.0000	C22—H22B	0.9900
C6—C7	1.520 (5)	C23—C24	1.523 (5)
C6—H6A	0.9900	C23—H23A	0.9900
C6—H6B	0.9900	C23—H23B	0.9900
C7—C8	1.534 (4)	C24—C25	1.495 (5)
C7—H7A	0.9900	C24—C28	1.555 (5)
C7—H7B	0.9900	C24—H24	1.0000
C8—C9	1.530 (4)	C25—C26	1.307 (6)
C8—C14	1.532 (4)	C25—C27	1.490 (5)
C8—H8	1.0000	C26—H26A	0.9500
C9—C11	1.497 (5)	C26—H26B	0.9500
C9—C10	1.520 (5)	C27—H27A	0.9800
C9—C19	1.557 (5)	C27—H27B	0.9800
C10—C19	1.474 (5)	C27—H27C	0.9800
C11—C12	1.534 (5)	C28—H28A	0.9800
C11—H11A	0.9900	C28—H28B	0.9800
C11—H11B	0.9900	C28—H28C	0.9800
C12—C13	1.520 (4)	C29—H29A	0.9800
C12—H12A	0.9900	C29—H29B	0.9800
C12—H12B	0.9900	C29—H29C	0.9800
C13—C18	1.547 (4)	C30—H30A	0.9800
C13—C17	1.553 (4)	C30—H30B	0.9800
C13—C14	1.561 (4)	C30—H30C	0.9800
C14—C15	1.536 (4)	C31—H31A	0.9800
C14—C32	1.547 (4)	C31—H31B	0.9800
C15—C16	1.537 (4)	C31—H31C	0.9800
C15—H15A	0.9900	C32—H32A	0.9800
C15—H15B	0.9900	C32—H32B	0.9800
C16—C17	1.551 (4)	C32—H32C	0.9800
C16—H16A	0.9900		
			
C29—O1—C3	113.6 (3)	C15—C16—H16B	110.3
C2—C1—C10	109.2 (3)	C17—C16—H16B	110.3
C2—C1—H1A	109.8	H16A—C16—H16B	108.6
C10—C1—H1A	109.8	C16—C17—C13	103.0 (2)
C2—C1—H1B	109.8	C16—C17—C20	112.1 (2)
C10—C1—H1B	109.8	C13—C17—C20	120.0 (3)
H1A—C1—H1B	108.3	C16—C17—H17	107.0
C3—C2—C1	110.7 (3)	C13—C17—H17	107.0
C3—C2—H2A	109.5	C20—C17—H17	107.0
C1—C2—H2A	109.5	C13—C18—H18A	109.5
C3—C2—H2B	109.5	C13—C18—H18B	109.5
C1—C2—H2B	109.5	H18A—C18—H18B	109.5
H2A—C2—H2B	108.1	C13—C18—H18C	109.5
O1—C3—C2	111.1 (3)	H18A—C18—H18C	109.5
O1—C3—C4	107.8 (3)	H18B—C18—H18C	109.5
C2—C3—C4	113.9 (3)	C10—C19—C9	60.1 (2)
O1—C3—H3	107.9	C10—C19—H19A	117.8
C2—C3—H3	107.9	C9—C19—H19A	117.8
C4—C3—H3	107.9	C10—C19—H19B	117.8
C31—C4—C3	109.2 (3)	C9—C19—H19B	117.8
C31—C4—C5	110.0 (3)	H19A—C19—H19B	114.9
C3—C4—C5	108.1 (3)	C22—C20—C21	110.0 (3)
C31—C4—C30	108.2 (4)	C22—C20—C17	111.1 (3)
C3—C4—C30	111.2 (3)	C21—C20—C17	110.7 (3)
C5—C4—C30	110.2 (4)	C22—C20—H20	108.3
C10—C5—C6	112.2 (3)	C21—C20—H20	108.3
C10—C5—C4	114.5 (3)	C17—C20—H20	108.3
C6—C5—C4	114.5 (3)	C20—C21—H21A	109.5
C10—C5—H5	104.7	C20—C21—H21B	109.5
C6—C5—H5	104.7	H21A—C21—H21B	109.5
C4—C5—H5	104.7	C20—C21—H21C	109.5
C7—C6—C5	110.4 (3)	H21A—C21—H21C	109.5
C7—C6—H6A	109.6	H21B—C21—H21C	109.5
C5—C6—H6A	109.6	C23—C22—C20	115.6 (3)
C7—C6—H6B	109.6	C23—C22—H22A	108.4
C5—C6—H6B	109.6	C20—C22—H22A	108.4
H6A—C6—H6B	108.1	C23—C22—H22B	108.4
C6—C7—C8	111.5 (3)	C20—C22—H22B	108.4
C6—C7—H7A	109.3	H22A—C22—H22B	107.4
C8—C7—H7A	109.3	C22—C23—C24	114.4 (3)
C6—C7—H7B	109.3	C22—C23—H23A	108.7
C8—C7—H7B	109.3	C24—C23—H23A	108.7
H7A—C7—H7B	108.0	C22—C23—H23B	108.7
C9—C8—C14	112.9 (2)	C24—C23—H23B	108.7
C9—C8—C7	112.1 (3)	H23A—C23—H23B	107.6
C14—C8—C7	114.1 (3)	C25—C24—C23	112.4 (3)
C9—C8—H8	105.6	C25—C24—C28	111.3 (3)
C14—C8—H8	105.6	C23—C24—C28	111.1 (3)
C7—C8—H8	105.6	C25—C24—H24	107.2
C11—C9—C10	117.5 (3)	C23—C24—H24	107.2
C11—C9—C8	118.3 (3)	C28—C24—H24	107.2
C10—C9—C8	118.6 (3)	C26—C25—C27	121.3 (4)
C11—C9—C19	118.3 (3)	C26—C25—C24	121.6 (4)
C10—C9—C19	57.2 (2)	C27—C25—C24	117.1 (3)
C8—C9—C19	112.3 (3)	C25—C26—H26A	120.0
C19—C10—C9	62.6 (3)	C25—C26—H26B	120.0
C19—C10—C1	114.4 (3)	H26A—C26—H26B	120.0
C9—C10—C1	119.6 (3)	C25—C27—H27A	109.5
C19—C10—C5	124.6 (4)	C25—C27—H27B	109.5
C9—C10—C5	120.0 (3)	H27A—C27—H27B	109.5
C1—C10—C5	109.3 (3)	C25—C27—H27C	109.5
C9—C11—C12	119.2 (3)	H27A—C27—H27C	109.5
C9—C11—H11A	107.5	H27B—C27—H27C	109.5
C12—C11—H11A	107.5	C24—C28—H28A	109.5
C9—C11—H11B	107.5	C24—C28—H28B	109.5
C12—C11—H11B	107.5	H28A—C28—H28B	109.5
H11A—C11—H11B	107.0	C24—C28—H28C	109.5
C13—C12—C11	113.5 (3)	H28A—C28—H28C	109.5
C13—C12—H12A	108.9	H28B—C28—H28C	109.5
C11—C12—H12A	108.9	O1—C29—H29A	109.5
C13—C12—H12B	108.9	O1—C29—H29B	109.5
C11—C12—H12B	108.9	H29A—C29—H29B	109.5
H12A—C12—H12B	107.7	O1—C29—H29C	109.5
C12—C13—C18	108.0 (3)	H29A—C29—H29C	109.5
C12—C13—C17	117.6 (3)	H29B—C29—H29C	109.5
C18—C13—C17	109.2 (3)	C4—C30—H30A	109.5
C12—C13—C14	109.2 (2)	C4—C30—H30B	109.5
C18—C13—C14	110.9 (2)	H30A—C30—H30B	109.5
C17—C13—C14	101.8 (2)	C4—C30—H30C	109.5
C8—C14—C15	114.7 (2)	H30A—C30—H30C	109.5
C8—C14—C32	110.2 (2)	H30B—C30—H30C	109.5
C15—C14—C32	107.5 (3)	C4—C31—H31A	109.5
C8—C14—C13	110.7 (2)	C4—C31—H31B	109.5
C15—C14—C13	102.3 (2)	H31A—C31—H31B	109.5
C32—C14—C13	111.2 (3)	C4—C31—H31C	109.5
C14—C15—C16	105.8 (2)	H31A—C31—H31C	109.5
C14—C15—H15A	110.6	H31B—C31—H31C	109.5
C16—C15—H15A	110.6	C14—C32—H32A	109.5
C14—C15—H15B	110.6	C14—C32—H32B	109.5
C16—C15—H15B	110.6	H32A—C32—H32B	109.5
H15A—C15—H15B	108.7	C14—C32—H32C	109.5
C15—C16—C17	107.1 (2)	H32A—C32—H32C	109.5
C15—C16—H16A	110.3	H32B—C32—H32C	109.5
C17—C16—H16A	110.3		
			
C10—C1—C2—C3	−60.8 (4)	C9—C11—C12—C13	5.2 (5)
C29—O1—C3—C2	66.9 (5)	C11—C12—C13—C18	−78.4 (4)
C29—O1—C3—C4	−167.6 (4)	C11—C12—C13—C17	157.4 (3)
C1—C2—C3—O1	−179.2 (3)	C11—C12—C13—C14	42.2 (4)
C1—C2—C3—C4	58.8 (4)	C9—C8—C14—C15	157.4 (2)
O1—C3—C4—C31	64.9 (4)	C7—C8—C14—C15	−73.0 (3)
C2—C3—C4—C31	−171.3 (3)	C9—C8—C14—C32	−81.1 (3)
O1—C3—C4—C5	−175.4 (3)	C7—C8—C14—C32	48.5 (3)
C2—C3—C4—C5	−51.6 (4)	C9—C8—C14—C13	42.3 (3)
O1—C3—C4—C30	−54.3 (5)	C7—C8—C14—C13	171.9 (2)
C2—C3—C4—C30	69.5 (5)	C12—C13—C14—C8	−68.0 (3)
C31—C4—C5—C10	169.8 (3)	C18—C13—C14—C8	50.9 (3)
C3—C4—C5—C10	50.6 (4)	C17—C13—C14—C8	167.0 (2)
C30—C4—C5—C10	−71.0 (4)	C12—C13—C14—C15	169.3 (3)
C31—C4—C5—C6	−58.5 (4)	C18—C13—C14—C15	−71.8 (3)
C3—C4—C5—C6	−177.7 (3)	C17—C13—C14—C15	44.3 (3)
C30—C4—C5—C6	60.7 (4)	C12—C13—C14—C32	54.8 (3)
C10—C5—C6—C7	−51.3 (4)	C18—C13—C14—C32	173.8 (3)
C4—C5—C6—C7	175.9 (3)	C17—C13—C14—C32	−70.1 (3)
C5—C6—C7—C8	65.7 (3)	C8—C14—C15—C16	−151.6 (3)
C6—C7—C8—C9	−52.5 (3)	C32—C14—C15—C16	85.5 (3)
C6—C7—C8—C14	177.5 (2)	C13—C14—C15—C16	−31.6 (3)
C14—C8—C9—C11	5.7 (4)	C14—C15—C16—C17	7.0 (3)
C7—C8—C9—C11	−124.9 (3)	C15—C16—C17—C13	20.6 (3)
C14—C8—C9—C10	158.6 (3)	C15—C16—C17—C20	150.9 (3)
C7—C8—C9—C10	28.0 (4)	C12—C13—C17—C16	−159.0 (3)
C14—C8—C9—C19	−137.7 (3)	C18—C13—C17—C16	77.5 (3)
C7—C8—C9—C19	91.7 (3)	C14—C13—C17—C16	−39.8 (3)
C11—C9—C10—C19	−107.5 (4)	C12—C13—C17—C20	75.7 (4)
C8—C9—C10—C19	99.4 (3)	C18—C13—C17—C20	−47.8 (4)
C11—C9—C10—C1	−3.5 (5)	C14—C13—C17—C20	−165.1 (2)
C8—C9—C10—C1	−156.6 (3)	C1—C10—C19—C9	−112.1 (3)
C19—C9—C10—C1	104.0 (4)	C5—C10—C19—C9	109.0 (4)
C11—C9—C10—C5	136.4 (3)	C11—C9—C19—C10	106.1 (4)
C8—C9—C10—C5	−16.7 (4)	C8—C9—C19—C10	−110.6 (3)
C19—C9—C10—C5	−116.0 (4)	C16—C17—C20—C22	54.8 (3)
C2—C1—C10—C19	−86.4 (4)	C13—C17—C20—C22	175.8 (3)
C2—C1—C10—C9	−157.6 (3)	C16—C17—C20—C21	177.3 (3)
C2—C1—C10—C5	58.6 (4)	C13—C17—C20—C21	−61.8 (4)
C6—C5—C10—C19	−47.9 (5)	C21—C20—C22—C23	67.8 (4)
C4—C5—C10—C19	84.9 (4)	C17—C20—C22—C23	−169.3 (3)
C6—C5—C10—C9	27.8 (4)	C20—C22—C23—C24	−179.6 (3)
C4—C5—C10—C9	160.6 (3)	C22—C23—C24—C25	60.3 (4)
C6—C5—C10—C1	171.5 (3)	C22—C23—C24—C28	−174.2 (3)
C4—C5—C10—C1	−55.7 (4)	C23—C24—C25—C26	−124.7 (4)
C10—C9—C11—C12	175.6 (3)	C28—C24—C25—C26	109.9 (4)
C8—C9—C11—C12	−31.2 (5)	C23—C24—C25—C27	56.3 (4)
C19—C9—C11—C12	110.0 (4)	C28—C24—C25—C27	−69.1 (4)
